# Solar-driven artificial hybrid systems for beyond natural biotransformation

**DOI:** 10.1093/nsr/nwag461

**Published:** 2026-07-30

**Authors:** Haotian Bai, Yan Zhang, Xu-Bing Li, Xiaoqiang Huang, Lu Yu, Xiao-Ning Guo, Weijian Chen, Yongjun Chen, Zijian Guo, Shu Wang, Yafei Dai

**Affiliations:** Key Laboratory of Organic Solids, Beijing National Laboratory for Molecular Sciences, Institute of Chemistry, Chinese Academy of Sciences, Beijing 100190, China; State Key Laboratory of Coordination Chemistry, Chemistry and Biomedicine Innovation Center (ChemBIC), School of Chemistry and Chemical Engineering, Frontier Interdisciplinary Science Research Center, Nanjing University, Nanjing 210023, China; Key Laboratory of Photochemical Conversion and Optoelectronic Materials, Technical Institute of Physics and Chemistry, Chinese Academy of Sciences, Beijing 100190, China; State Key Laboratory of Coordination Chemistry, Chemistry and Biomedicine Innovation Center (ChemBIC), School of Chemistry and Chemical Engineering, Frontier Interdisciplinary Science Research Center, Nanjing University, Nanjing 210023, China; State Key Laboratory of Advanced Environmental Technology, School of Environment, University of Science and Technology of China, Hefei 230026, China; Key Laboratory of Photochemical Conversion and Optoelectronic Materials, Technical Institute of Physics and Chemistry, Chinese Academy of Sciences, Beijing 100190, China; Key Laboratory of Organic Solids, Beijing National Laboratory for Molecular Sciences, Institute of Chemistry, Chinese Academy of Sciences, Beijing 100190, China; Department of Interdisciplinary Sciences, National Natural Science Foundation of China, Beijing 100085, China; State Key Laboratory of Coordination Chemistry, Chemistry and Biomedicine Innovation Center (ChemBIC), School of Chemistry and Chemical Engineering, Frontier Interdisciplinary Science Research Center, Nanjing University, Nanjing 210023, China; Key Laboratory of Organic Solids, Beijing National Laboratory for Molecular Sciences, Institute of Chemistry, Chinese Academy of Sciences, Beijing 100190, China; Department of Interdisciplinary Sciences, National Natural Science Foundation of China, Beijing 100085, China

**Keywords:** natural–artificial integration, biohybrid systems, efficient biosynthesis, artificial photosynthesis, sustainable energy and chemicals

## Abstract

Biotransformation plays a crucial role in addressing global challenges related to resource utilization and energy sustainability. Natural processes, such as photosynthesis and microbial metabolism, transform external resources into functional compounds. However, these processes are often constrained by inefficiencies, slow reaction rates and limited product diversity. Recent advancements across the physical, chemical and biological sciences have led to the convergence of natural and artificial strategies that effectively tackle these challenges. These developments, alongside progress in artificial photosynthesis, offer solutions for CO_2_ reduction, N_2_ fixation and sustainable chemical production, thereby broadening the scope of biotransformation beyond traditional natural processes. Herein, we first introduce recent representative achievements in resolving the structures and catalytic mechanisms of proteins/protein complexes/enzymes involved in natural biotransformation processes. Our review then highlights natural photosynthesis-inspired organic–biological hybrid systems, focusing on the conversion of CO_2_ and N_2_ as well as the diverse range of value-added products they enable. Furthermore, this paper will explore the potential of artificial photosynthesis systems, examining their integration and applications in advancing biotransformation processes. Finally, we discuss key challenges and propose future strategies to advance research in this field. The ongoing advancements in artificial systems hold immense promise for revolutionizing and paving the way for a sustainable future that extends beyond natural biotransformation.

## Teaser

Solar-driven artificial hybrid systems integrate natural and artificial processes to overcome the intrinsic limitations of natural biotransformation, enabling sustainable production of fuels, chemicals and other high-value products.

## INTRODUCTION

Biotransformation, in addition to natural synthesis, plays a crucial role in evolution, ecological balance and technological progress [1–6]. In nature, biochemical networks within biological systems convert external resources, such as inorganic carbon, nitrogen sources and biomass, into functional compounds [7–11]. While traditional natural synthesis processes have their value, they often suffer from limitations such as low yields, slow rates and restricted product diversity [12–14]. For instance, plants undergo photosynthesis to convert carbon dioxide and water into organic compounds like starch, while releasing oxygen. Despite its importance in addressing resource and energy challenges, photosynthesis is remarkably inefficient (∼1%) due to the limited types of pigments and light-harvesting capacity [15,16]. On the other hand, microorganisms, such as bacteria, yeasts and algae, offer higher efficiency and versatility in biotransformation, allowing for the rapid production of complex natural products. However, biotransformation using these microorganism systems still faces obstacles such as low yields, metabolic strain and technological challenges [17–20]. To overcome and address these limitations, it is necessary to explore innovative strategies beyond natural biotransformation that enable scalable and stable production of the target compounds.

Rapid advancements in the fields of science and technology have resulted in the development of a variety of innovative methods for highly efficient biotransformation that extend beyond natural processes [21,22]. Recent achievements in physical and chemical technologies, including ultrafast time-resolved X-ray free-electron lasers (XFELs) and cryo-electron microscopy (cryo-EM), have provided deeper insights into the critical phases and mechanisms of natural biotransformation [21,23–25]. This has facilitated a comprehensive analysis of processes such as matter transformation and energy transfer. Furthermore, advancements in biological sciences, such as omics technologies and gene editing, have offered valuable tools for reengineering metabolic pathways and customizing biosynthetic functions. Additionally, the integration of natural and artificial investigation has the potential to enhance energy conversion efficiency significantly. For instance, the integration of artificial materials with cells and other biological systems overcomes the limitations of natural photosynthesis and microbial biotransformation by improving light absorption, charge separation and spectral adaptability. By combining insights from natural biotransformation with hybrid systems of chemical materials and natural cells, innovative pathways for highly efficient biotransformation are emerging [12,26–30]. Significant advancements moreover have been made in the field of artificial photosynthesis (AP) through enhancements in catalyst design, system architecture and understanding of underlying mechanisms. These developments promote the efficiency of processes such as water splitting, CO_2_ reduction and nitrogen fixation [31–35]. These advancements pave the way for progress that transcends traditional strategies.

**Figure 1.**
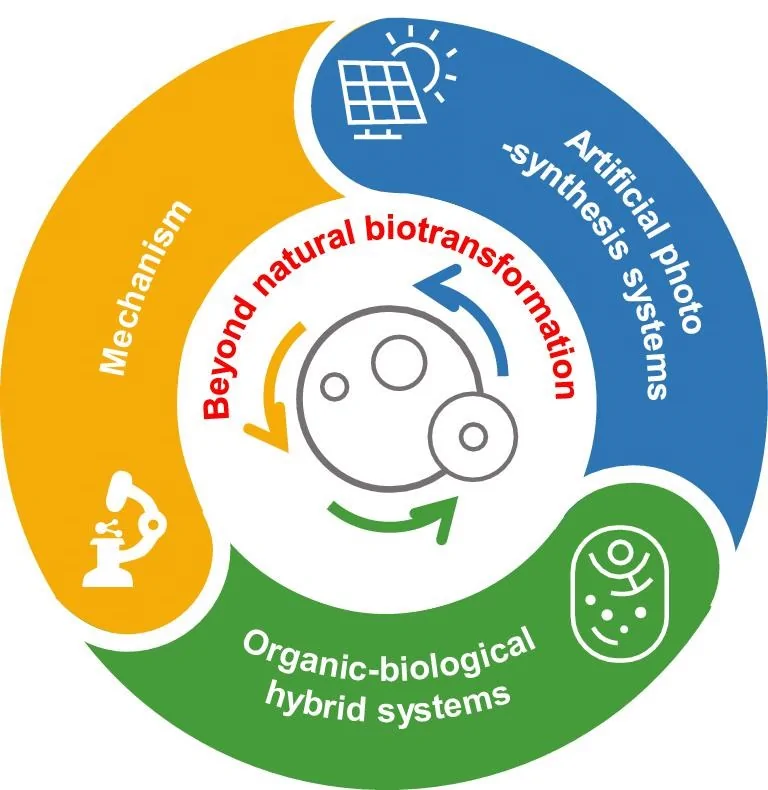
Schematic overview of solar-driven artificial hybrid systems for beyond natural biotransformation.

In this review, we will commence by examining recent representative advances in resolving the high-resolution structures and catalytic mechanisms for components in natural biotransformation processes. Subsequently, we will focus on natural photosynthesis-inspired organic–biological hybrid systems designed for biotransformation. Furthermore, our discussion will extend to AP systems and their applications in material transformation. Finally, we will identify key challenges prevalent in current research and offer perspectives regarding potential solutions and future directions in this field (Fig. 1).

## STRUCTURAL AND MECHANICAL FOUNDATIONS FOR RATIONAL HYBRID DESIGN

### Structural analysis of photosynthetic proteins/protein complexes

The solar-driven artificial hybrid systems have the potential to achieve biotransformation efficiencies that surpass natural processes, presenting opportunities for efficient biosynthesis of high-value compounds. Nevertheless, this objective can only be achieved with a comprehensive understanding of both the structure and functional mechanisms of the proteins/protein complexes involved in natural photosynthesis processes as well as a profound knowledge of the catalytic mechanisms of key enzymes involved in natural biotransformation processes. Understanding the precise architectures of natural photosynthetic machineries provides essential blueprints for overcoming their inherent limitations. This section examines key structural advances, explicitly highlighting how these insights inform the design of artificial and hybrid systems described in subsequent sections.

Recent advances in single-particle cryo-EM, XFEL and other techniques have unveiled unprecedented structural and catalytic details concerning the proteins/protein complexes involved in photosynthesis, thus providing thorough insights into key steps in photosynthesis, such as light harvesting, charge separation, energy transformation and photoprotection (Fig. 2a). Meanwhile, recent advances in single-particle cryo-EM have provided unprecedented structural information about these huge membrane complexes. Currently, the overall structures of two key photosynthetic systems, photosystem I (PSI, plastocyanin-ferredoxin oxidoreductase) and photosystem II (PSII, water-plastoquinone oxidoreductase) [36–38], along with their core reaction centers and peripheral antenna systems [light-harvesting complex I (LHCI) for PSI and light-harvesting complex II (LHCII) for PSII], [39,40] have been resolved. Moreover, the structures of supercomplexes [25,41] and megacomplexes [9,42–44] formed by further assembly of these proteins/protein complexes have also been clarified, providing direct structural information for understanding the electron and energy transfer pathways within the giant molecular machinery of photosynthesis. Beyond structural biology techniques, an increasing number of analytical methods have been employed to reveal the physicochemical mechanisms at the molecular level in photosynthesis, such as advanced femtosecond broadband transient fluorescence spectroscopy, femtosecond fluorescence conical optical parametric amplification spectroscopy, two-dimensional electronic spectroscopy and two-dimensional electronic vibrational spectroscopy, along with density functional theory calculations, which have provided more profound insights into the mechanisms of light harvesting [45,46], charge separation [47,48], energy transformation [49] and photoprotection [10,50].

**Figure 2.**
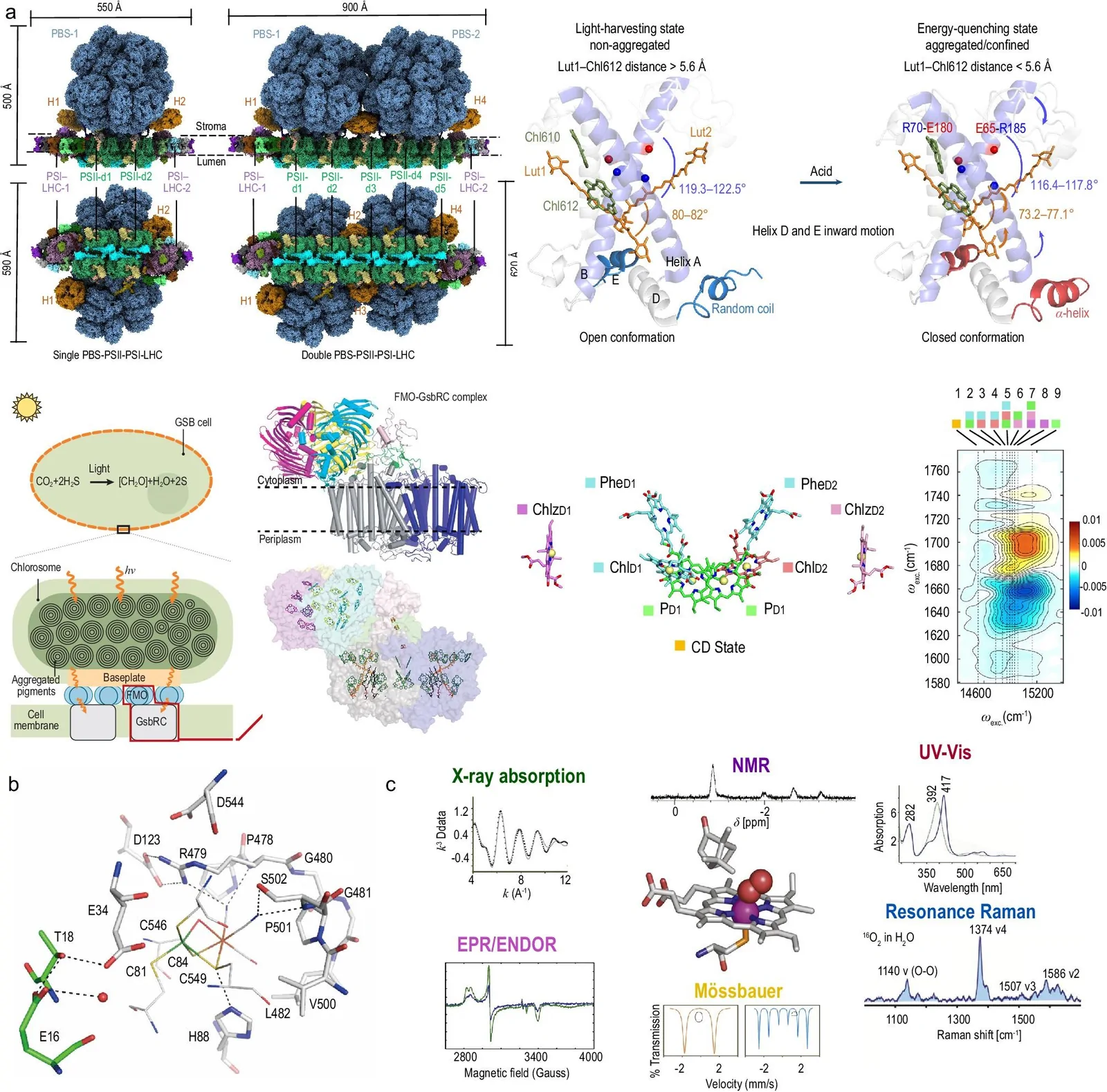
(a) Structural and mechanistic studies of protein/protein complexes in natural photosynthesis. Adapted with permission from [42,46,48,49]. Copyright 2023, Springer Nature. Copyright 2020, American Association for the Advancement of Science. Copyright 2023, Springer Nature. (b) Structural and mechanistic studies of enzymes involved in natural biotransformation processes. Adapted with permission from [61]. Copyright 2022, American Chemical Society. (c) Some exampled methods for the mechanistic investigation. Adapted with permission from [69]. Copyright 2018, Elsevier.

The structural elucidation of PSII’s oxygen-evolving complex (OEC) and the energy transfer pathways in PSI–LHCI/LHCII supercomplexes provide critical blueprints for addressing key bottlenecks in AP. Specifically, the precise spatial arrangement of chlorophylls in LHCII has inspired the design of artificial antenna materials with optimized Förster resonance energy transfer efficiencies to enhance chloroplast light harvesting [51]. Furthermore, the revelation that the Mn_4_CaO_5_ cluster in PSII achieves water oxidation with minimal overpotential despite operating at neutral pH has directly guided the development of molecular water oxidation catalysts. For instance, the di-μ-oxo-bridged Mn- or Co-based complexes developed by Sun and Zhang explicitly mimic the cubane-like geometry and proton-coupled electron transfer (PCET) mechanism of the OEC, achieving remarkably improved catalytic rates compared to unstructured metal oxides [52,53]. These examples underscore how high-resolution structural insights enable the rational replication of natural catalytic sites in synthetic systems.

### Catalytic mechanisms of proteins/protein complexes involved in biotransformation

On the other hand, to establish solar-driven artificial hybrid systems that surpass the catalytic efficiency of natural biotransformation processes, it is essential to gain an in-depth understanding of the structure and mechanisms of key enzymes responsible for the core catalytic transformations in natural bioconversion systems [54,55]. Here, we briefly summarize some of the latest progress in catalytic mechanisms studies of ribulose-1,5-bisphosphate carboxylase/oxygenase (RuBisCO), nitrogenase, hydrogenase and cytochrome P450 (P450), each of which catalyzes the conversion of natural small molecules (e.g. CO_2_, N_2_ and H_2_) or engages in multiple reactions.

Among the six CO_2_ fixation pathways found in nature, the Calvin cycle, mediated by RuBisCO, is the most important [56,57]. While structural biology has provided static snapshots of key biotransformation machineries, several ongoing controversies highlight gaps in our understanding that limit rational artificial system design. Regarding RuBisCO assembly, the precise biophysics of carboxysome formation remains debated—whether the liquid-like condensates induced by carbon concentrating mechanism protein M (CcmM), a carboxysome scaffolding protein, form through passive liquid–liquid phase separation or require active, redox-regulated remodeling involving disulfide bond dynamics is not fully resolved. Recently, cryo-EM together with biochemical assays revealed both the high-resolution structure and a rather dynamic structural intermediate of a 32-subunit RbcL8_8_Raf1_8_RbcX_16_ assembly, providing a model on how chaperones work in concert to facilitate and regulate cyanobacterial RuBisCO assembly as well as disassembly of RuBisCO condensates [58,59]. This knowledge of RuBisCO’s conformational changes during catalysis has informed the development of protective scaffolds in polymer–bacteria hybrids, where the poly(fluorene-co-phenylene) (PFP)/perylene diimide derivative (PDI) heterojunction not only replaces natural light harvesting but also creates localized chemical environments that mimic the concentrating mechanism of carboxysomes, achieving quantum yields exceeding natural photosynthesis under certain conditions [60]. Hydrogenases are hydrogen-reducing metalloenzymes found in microorganisms, where they catalyze the oxidation of hydrogen or the reduction of protons, thus participating in microbial energy metabolism. In some organisms, hydrogenases are also involved in hydrogen uptake, hydrogen sensing and carbon dioxide fixation (Fig. 2b) [61]. For example, in the case of [FeFe]-hydrogenase, through a combination of various methods and the integration of multiple methodologies, including electron paramagnetic resonance (EPR), structural determination, Fourier transform infrared spectroscopy, mass spectrometry, cell-free biosynthesis and spectroscopy, significant progress has been made in understanding the molecular mechanism of [FeFe]-hydrogenase [62]. Research into the biosynthesis and catalytic mechanisms of its active site has provided a theoretical foundation for efficiently and effectively mimicking the function of hydrogenases under mild conditions [63]. Nitrogenase is a complex metalloenzyme that catalyzes the conversion of atmospheric N_2_ into biologically available NH_3_, a crucial step in the global nitrogen cycle. The functional significance and complexity of nitrogenase have inspired generations of researchers to conduct extensive studies on its mechanisms. Recent advances in structural biology have significantly improved the resolution of nitrogenase, enabling the identification of active-site structures and key catalytic atoms [64–66]. These studies, together with rapid freeze-quench techniques combined with EPR, electron nuclear double resonance (ENDOR) and electron spin echo envelope modulation (ESEEM) spectroscopy, have allowed the capture and characterization of multiple catalytic intermediates, providing important insights into the electronic structure and reaction pathway of nitrogenase [7,8]. However, several key mechanistic questions remain unresolved. The electron transfer (ET) pathway within nitrogenase is still under debate. The ‘deficit-spending’ model, in which the P-cluster first reduces FeMoco followed by rapid electron replenishment from the Fe protein, differs fundamentally from the conventional sequential ‘hopping’ mechanism. In addition, the atomic-level ET route from the P-cluster to FeMoco remains unclear, as does the exact chemical identity of late-stage intermediates during N_2_ reduction, including diazene- or hydrazine-like species. These uncertainties limit the rational design of artificial nitrogen fixation systems, as current biohybrid approaches still rely largely on empirical electron delivery strategies rather than mechanism-guided optimization.

Lastly, P450 enzymes are heme-containing enzymes widely distributed in microorganisms, plants, animals and humans. They are among the most versatile biocatalysts in nature, capable of catalyzing a wide range of reactions, including oxidation, epoxidation, hydroxylation and demethylation [67]. Currently, various spectroscopic methods, such as ultraviolet-visible absorption, EPR, nuclear magnetic resonance, ENDOR, Mössbauer, X-ray absorption and resonance Raman, have been applied to provide multifaceted and detailed mechanistic insights into the biophysics and biochemistry of these fascinating enzymes (Fig. 2c). The combination of these spectroscopic techniques with novel approaches, such as cryo-reduction and nanodisc technology, has enabled the generation, trapping and characterization of long-sought transient intermediates [68,69]. Moreover, the comprehensive mechanistic understanding of P450 catalysis—particularly the identification of transient intermediates—has established a mechanistic framework for designing artificial photoenzymes. Specifically, understanding that P450s rely on controlled radical chemistry within a hydrophobic pocket inspired the design of enzymatic electron–donor–acceptor (EDA) complexes that generate substrate radicals upon photoexcitation [70–72].

### Creation and functionalization of artificial photoenzymes

The integration of biocatalysis and photocatalysis harnesses the benefits of both processes, offering several advantages, such as new-to-nature reactivity, otherwise unattainable selectivity and enhanced environmentally friendly synthesis [11,73,74]. However, the number of naturally occurring photoenzymes is limited. Recent advancements have paved the way for hybrid systems that enable beyond natural synthesis through visible-light illumination of natural enzymes. In this section, several strategies have been explored (Fig. 3a), including the illumination of enzymatic EDA complexes, direct excitation of flavin-dependent enzymes and the construction of artificial enzymes inserted with light-absorbing antennae.

**Figure 3.**
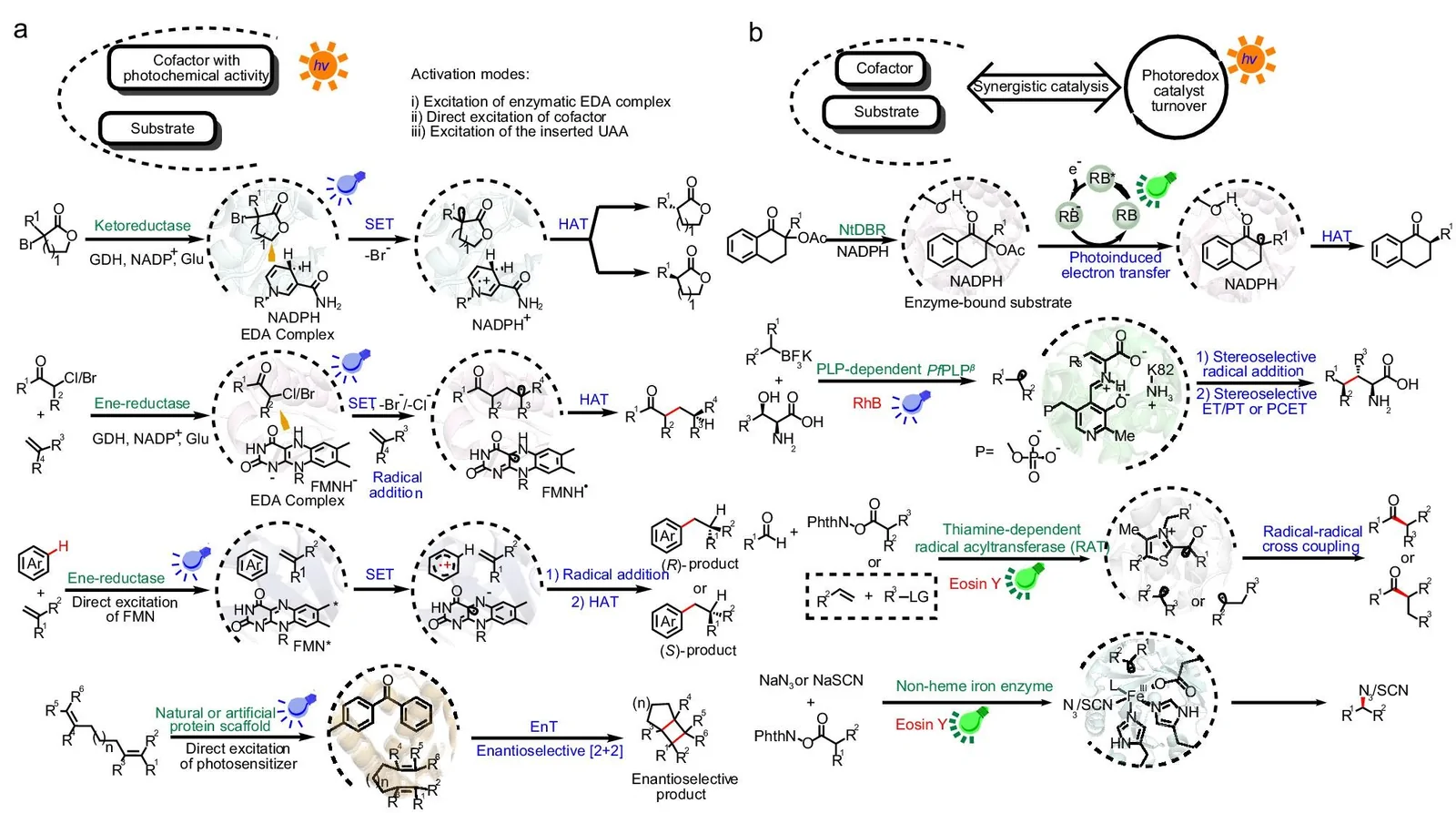
(a) Creation and functionalization of artificial photoenzymes. (b) Dual photo/biocatalytic strategy for new-to-nature biotransformation.

An enzymatic EDA complex formed between a reduced cofactor (such as nicotinamide adenine dinucleotide phosphate for ketoreductases or hydroquinone flavin for ene-reductases) and an electron-deficient unnatural substrate within the enzyme’s active site, can be excited by visible light, thereby initiating unnatural radical processes to unlock biotransformation that go beyond natural capabilities. In 2016, Hyster and co-workers reported a photoinduced enantioselective dehalogenation reaction catalyzed by nicotinamide-dependent ketoreductase, which was the first example of unlocking new-to-nature enzyme reactivity with visible light [70]. In 2020, the Zhao group irradiated ene-reductases to enable an intermolecular hydroalkylation of styrene with *α*-halo carbonyl advancing the field to intermolecular biotransformations [71]. Recently, Hyster and coworkers achieved an enantioselective C*sp*^3^–C*sp*^3^ cross-electrophile coupling reaction between alkyl halides and nitroalkanes [27]. Zhao’s group accomplished asymmetric incorporation of fluorinated motifs into vinyl arenes [72]. Very recently, Huang and co-workers repurposed engineered nicotinamide-dependent imine reductases as a new category of visible-light-activable enzymes, facilitating biocatalytic modular synthesis of chiral amines [26].

Quinoid-state flavins are natural enzyme cofactors that exhibit a maximum absorption peak of ∼450–460 nm. The flavin adenine dinucleotide (FAD)-dependent fatty acid photodecarboxylase from *Chlorella variabilis* (*Cv*FAP), first identified in 2017 by Beisson and co-workers [75], catalyzes the decarboxylation of medium-to-long-chain fatty acids to produce the corresponding alkanes or alkenes. Mechanistic studies revealed that the cofactor FAD can be excited by visible light to initiate the single-electron oxidation of fatty acid anions [76]. In 2021, Wu *et al*. achieved the first asymmetric unnatural reaction using an engineered *Cv*FAP [77]. Under blue light illumination, the mutant *Cv*FAP_G462Y facilitated the selective photodecarboxylation of *α*-amino acids and *α*-hydroxy acids with (*S*)-configurations, yielding unreacted (*R*)-products with enantiomeric excesses of up to 99%. In 2024, Yang and co-workers engineered *Cv*FAP to catalyze an unnatural photoredox intramolecular radical cyclization [78]. Drawing inspiration from the direct excitation of flavin cofactor of *Cv*FAP, Huang and co-workers leveraged the highly oxidizing property of excited flavins to catalyze an abiological radical hydroarylation building C*sp*^2^–C*sp*^3^ bonds in an enantiodivergent manner in 2023 [79].

Introducing light-active unnatural amino acids into protein scaffolds represents another approach to constructing artificial photoenzymes. In 2018, the Wang group developed a genetically encoded photosensitizer protein for the reduction of CO_2_ in synergy with an artificial metal center [80]. Notably, they clearly demonstrated that the distance between the genetically inserted chromophore and the catalytically active metal center plays a critical role in CO production. Maximum efficiency was achieved at a distance of 11.9 Å, with shorter or longer distances resulting in reduced activity, suggesting an optimal distance for ET. Subsequently, in 2022, the groups of Green [81] and Wu [82], respectively, achieved advancements in creating artificial photoenzymes through the genetic incorporation of 4-benzoylphenylalanine into protein scaffolds and demonstrated their application in enantioselective [2 + 2] cycloadditions. More recently, Xiang’s group realized the genetic incorporation of thioxanthone-containing amino acids into the LmRr protein to furnish an asymmetric photocycloaddition [83].

### Dual photo/biocatalytic strategy for new-to-nature biotransformations

To address the challenge associated with enzymes’ limited photophysical properties, exogenous photocatalysts can be incorporated to work synergistically with enzymes. This strategy has emerged as a robust and modular approach. So far, nicotinamide-, flavin-, pyridoxal 5′-phosphate (PLP)- and thiamine-dependent enzymes, as well as non-heme iron enzymes (Fig. 3b), most of which are not visible-light activatable in their natural states, have been demonstrated to be capable of harnessing visible-light-induced radical processes for the new-to-nature biosynthesis.

In 2018, through the combination of nicotinamide-dependent double-bond reductase NtBDR and the photocatalyst Rose Bengal, the Hyster group introduced a synergistic dual catalytic system that enabled unnatural enantioselective deacetylation [84]. The excited-state Rose Bengal initiated a single-electron transfer (SET) process of the enzyme-bound α-acetoxy ketones, resulting in the formation of ketyl radical intermediates within the active site after the acetate anion was eliminated. Subsequent hydrogen-atom transfer (HAT) with nicotinamide adenine dinucleotide phosphate hydrogen (NADPH) in the enzyme pocket led to the generation of the final products. In the same year, Hartwig and co-workers reported a cooperative catalytic system that combined photosensitized isomerization with enzymatic reduction by integrating an ene-reductase and a photocatalyst [85]. Through energy transfer from excited photocatalysts, *Z* alkenes were transformed into *E* alkenes. Simultaneously, the ene-reductases worked together to catalyze the asymmetric reduction of the C=C bond of the more reactive *E* isomers. In 2023, Yang and co-workers introduced a dual-catalytic system that combined photocatalysis with PLP biocatalysis to generate valuable non-canonical amino acids [86]. The excited-state Rhodamine B facilitated the SET oxidation of benzyltrifluoroborate salts, leading to the formation of alkyl radical intermediates. Meanwhile, the engineered enzyme converted *β*-hydroxy-*α*-amino acids into electrophilic aminoacrylates through a PLP-catalyzed pathway. These two active intermediates subsequently underwent a radical addition within the enzyme’s active site, resulting in the formation of an azaallyl radical intermediate. The final ET/proton transfer or PCET ultimately led to the target products through hydrolysis. In 2023, Huang and co-workers unveiled a dual photobiocatalytic system that combined organic dye Eosin Y with an engineered thiamine pyrophosphate (ThDP)-dependent enzyme [87]. Leveraging the synergy of photoredox catalysis and directed evolution, ThDP-dependent enzymes were reshaped to enable unnatural and highly enantioselective radical acylation. The electron-rich Breslow intermediate bound to the enzyme underwent single-electron oxidation by photoexcited Eosin Y* and led to a ThDP-derived ketyl radical. Concurrently, Eosin Y^•^**^—^** reduced the radical precursor, resulting in a prochiral benzylic radical. Ultimately, radical-radical cross-coupling occurred in the enzyme’s active site, forming the C*sp*^2^–C*sp*^3^ bond in an enantioselective manner, and releasing the radical acyl transferase (RAT) for subsequent cycles. In 2024, Huang and co-workers further applied this dual catalysis to achieve a challenging photobiocatalytic enantioselective three-component radical cross-coupling [88]. Very recently, Huang and co-workers introduced a biocatalytic platform that integrates photoredox and metalloenzymatic catalysis for enantioselective radical transformations [89]. When illuminated with green light, the Eosin Y photocatalyst activated natural non-heme iron enzymes to facilitate enantioselective decarboxylative azidation and thiocyanation of *N*-hydroxyphthalimide esters.

Despite significant breakthroughs in expanding new chemical space, photobiocatalysis still suffers from several challenges. For instance, enzyme instability under illumination—particularly for photoenzymes that require near-ultraviolet or violet light—and incompatibility with exogenous photocatalyst, often result in efficiency and productivity inferior to those of natural enzymes. Additionally, optimizing dual photo- and biocatalysts simultaneously while achieving matched kinetics introduces further complexity. Addressing these issues will require integrated strategies across multiple disciplines and will be essential for the practical application of unnatural photobiosynthesis.

## PHOTOSYNTHESIS-INSPIRED ORGANIC–BIOLOGICAL HYBRID SYSTEMS FOR BIOTRANSFORMATION PROCESSES

### Advancing natural photosynthesis and artificial photosynthetic systems for green synthesis

Enzymatic catalysis offers high specificity and turnover rates with minimal side products, but is often limited by poor stability and dependence on external cofactors. In contrast, whole-cell biocatalysts provide system-level metabolic networks that confer distinct advantages, such as autonomous cofactor regeneration, enhanced robustness and the coordinated regulation of complex biosynthetic pathways. However, their performance is still constrained by intrinsic metabolic and energetic limitations. Inspired by natural photosynthesis, integrating synthetic light-harvesting materials with living cells provides a strategy to overcome these bottlenecks. Such organic–biological hybrids allow precise control of electron flow and metabolism, enhancing both the efficiency and sustainability of biotransformation. The design of these systems requires seamless integration of light harvesting, energy conversion and biological catalysis, with energetic alignment between semiconductor band gaps and the redox potentials of target intermediates to ensure sufficient driving force. Optimizing the biotic–abiotic interface is also essential to facilitate efficient charge transport while preserving cellular integrity.

As a limitation of natural photosynthesis, more than half of the sun’s energy cannot be utilized by photosynthetic organisms like cyanobacteria, algae and green plants as it falls outside their absorption range. Therefore, the development of innovative materials that can efficiently capture light is necessary to enhance the efficiency of photosynthesis. One such material is conjugated polymer nanoparticles (CPNs), which serve as artificial antennas. These CPNs can enhance the light capture by chloroplasts, accelerate the electron transport within PSII and augment the conversion of solar energy in the chloroplast/CPN complexes [51]. By employing a comparable strategy, the red-emitting poly(boron-dipyrromethene-co-fluorene) (PBF) effectively regulates photosynthetic state transitions between PSI and PSII, which could enhance light-independent reactions and lead to faster growth and earlier flowering in *Arabidopsis thaliana* (Fig. 4a) [12]. Furthermore, a gold nanocluster/organic semiconductor heterostructure referred to as AuNC@OFTF has been developed to engage in the intracellular electron transport chain of live cyanobacteria, which boosts cyanobacterial photosynthesis by introducing external electrons and leads to a 5-fold increase in photocurrent. This utilization of advanced artificial photoelectronic materials presents a promising strategy to optimize photosynthetic pathways [90]. All the exampled biohybrid systems integrating light-trapping materials with photosynthetic organisms effectively convert solar energy into storable chemical energy, offering significant potential for sustainable biotransformation and chemical production.

**Figure 4.**
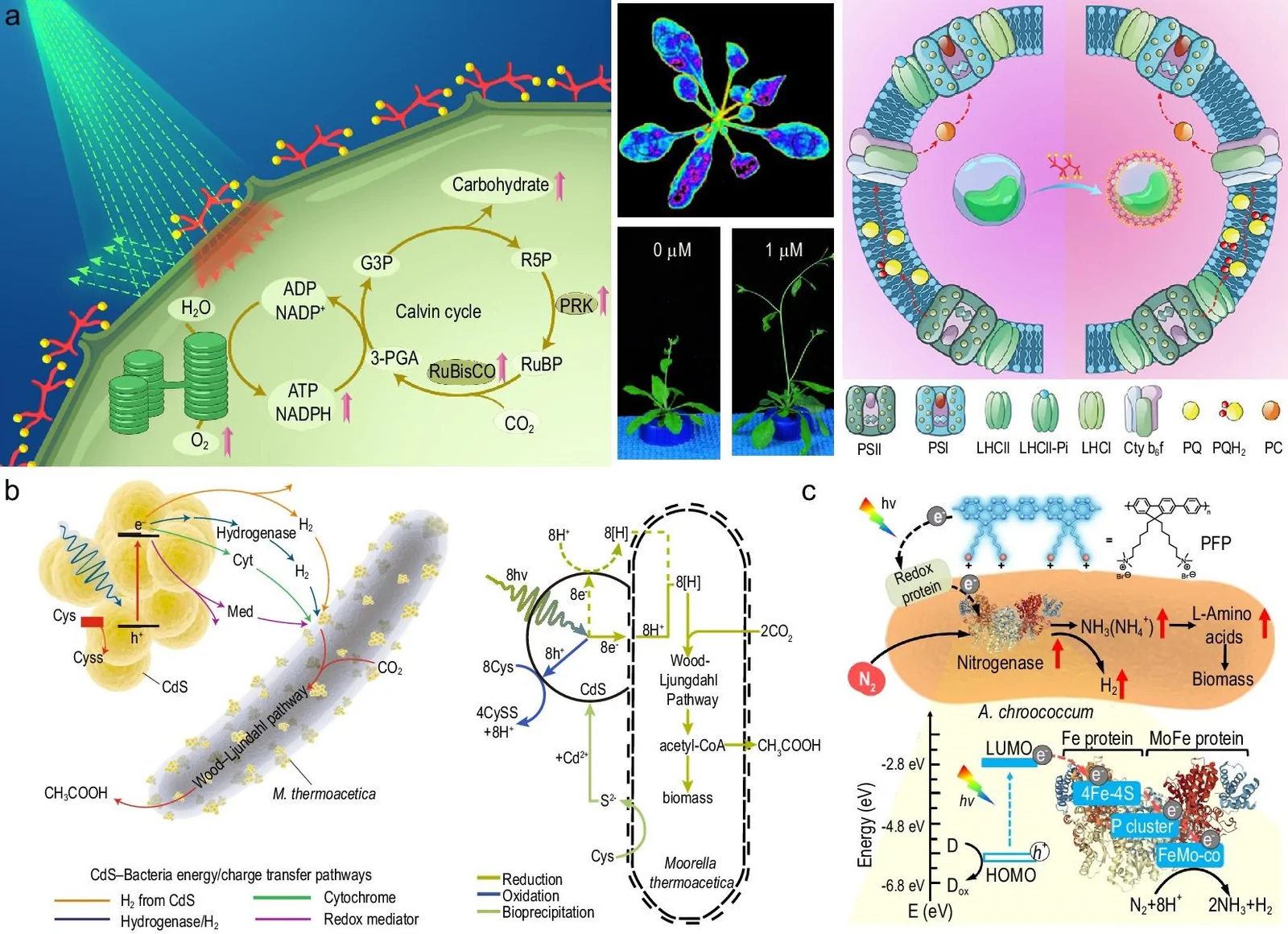
(a) The red-emitting PBF efficiently regulates photosynthetic state transitions between PSI and PSII in *A. thaliana.* Adapted with permission from [12]. Licensed under CC BY 4.0. (b) The CdS nanoparticles were initially utilized with non-photosynthetic *M. thermoacetica* by injecting external photoelectrons to generate acetic acid from CO_2_. Adapted with permission from [20,22]. Copyright 2016, American Association for the Advancement of Science. Copyright 2018, Springer Nature. (c) The biohybrid system for artificially photosynthetic N_2_ fixation by PFP and the non-photosynthetic *Azotobacter chroococcum*. Adapted with permission from [91]. Copyright 2023, John Wiley and Sons.

Microorganisms possess intricate metabolic networks and programmable synthetic pathways, enabling them to produce a variety of target products. If non-photosynthetic microorganisms could achieve photosynthetic autotrophy, they could potentially utilize renewable resources such as air, water and sunlight to generate chemicals efficiently. However, the conversion and storage of solar energy as chemical energy, exemplified by the production of acetic acid from CO_2_, presents a significant research challenge. Drawing inspiration from natural photosynthetic systems, researchers have utilized specific artificial materials that demonstrate strong light absorption and bacterial biocompatibility as light-capturing antennas. These materials are coupled with non-photosynthetic bacterial cells. For instance, cadmium sulfide (CdS) nanoparticles were initially utilized with non-photosynthetic *Moorella thermoacetica* (Fig. 4b) [20]. Through the intracellular injection of external photoelectrons, this self-photosensitizing system successfully generated acetic acid from CO_2_ when subjected to white light irradiation. As one of the early studies on biohybrid systems composed of artificial nanomaterials and biological cells, the authors carefully observed the correlation between extracellular photoelectron and intracellular reducing equivalents ([H]). However, the potential mechanisms of ET across the biological interface were not discussed in detail. Organic semiconducting polymers, particularly *π*-conjugated molecules, hold great promise for biohybrid systems due to their biocompatibility, tunable energy levels, photoelectric properties and variable band gaps. The Wang group developed a p–n heterojunction composed of PFP and a PDI to enable artificial photosynthetic functionality in non-photosynthetic bacteria. This system, characterized by the high photoelectric conversion efficiency of PFP/PDI and efficient ET at the biointerface, successfully activated the Wood–Ljungdahl pathway in *M. thermoacetica* under illumination, leading to acetate production with a quantum yield of 1.6%. To further investigate the ET mechanism, the redox properties of *M. thermoacetica* were characterized by cyclic voltammetry. The results revealed specific redox peaks at −0.2 V and a reduction peak at −0.5 V, which were assigned to membrane-bound proteins such as rubredoxins (Rd) and flavoproteins (Fp), consistent with previous reports. Since the reduction potential of the PDI/PFP heterojunction is more negative than those of Rd and Fp, it was suggested that the system provides a sufficient driving force for the efficient and direct transfer of photoexcited electrons across the bacterial membrane [60].

Nitrogen (N_2_) fixation is a crucial process in global biogeochemical cycles. The extensively used Haber–Bosch process, employed on an industrial scale, has encountered criticism owing to its high temperature and pressure requirements, elevated energy consumption and generation of CO_2_ emissions. Although biological N_2_ fixation under ambient conditions is more environmentally friendly, its practical application is hindered by the low efficiency and oxygen sensitivity of nitrogenase. To address these challenges, King and co-workers developed a photochemical model for artificial N_2_ fixation by creating a biohybrid system consisting of CdS nanorods and the MoFe protein in nitrogenase, which facilitates light-induced nitrogen reduction to ammonia. The authors proposed that photogenerated electrons from the CdS nanorods could effectively mimic the role of electrons typically derived from natural ATP hydrolysis. By directly activating the nitrogenase reaction center, these high-energy electrons facilitate light-induced N_2_ reduction [19]. Building upon the significant progress achieved with semiconductor materials that possess efficient light conversion properties for driving biosynthetic processes, Wang’s group introduced a novel biohybrid system for artificial photosynthetic N_2_ fixation, which utilized conjugated polymers (PFP) and the non-photosynthetic *A. chroococcum* (Fig. 4c). By leveraging a coordinated photogenic electron transport pathway across biological interfaces, which was facilitated by cell surface oxidized proteins, external photoelectrons improved the N_2_ fixation capacity of the MoFe protein within living bacterial cells. Notably, this system utilized the by-products of biological culture and metabolism as electron sacrificial agents, which eliminated the need for external sources to improve the atomic economy [91].

In the absence of direct electron transport proteins, the utilization of artificial electron shuttles can enhance the transfer of photogenerated electrons within cells, leading to improved energy utilization and biosynthetic efficiency. For instance, the groups of Wang and Tian demonstrated the incorporation of neutral red as an electron shuttle in a hybrid system that coupled organic semiconductor polymer dots with *Ralstonia eutropha* H16. This innovative approach achieved efficient conversion of CO_2_ into poly-3-hydroxybutyrate (PHB) [92]. These advancements signify the development of artificial photosynthetic systems that integrate artificial materials with biological metabolism. Moreover, this strategy offers an environmentally friendly and customizable method for harnessing renewable resources in biotransformation processes.

### Advancing biohybrid systems for highly efficient biosynthesis and broaden applications

The utilization of artificial materials to confer desired functionalities to natural microbial cells through optical, electrical or chemical energy process control represents a significant advancement in scientific research. Nevertheless, it is crucial to improve the efficiency of these systems while maintaining their inherent advantages in order to enable broader applications. The investigation conducted by the Liu group using *Sporomusa ovata* cells demonstrated that the injection of external photoelectrons more effectively alters biological metabolism and enhances biotransformation compared to the introduction of external substances such as hydrogen [23]. Yang’s group has investigated the previously unknown electron transport mechanism from semiconductors to bacteria using transient absorption and time-resolved infrared spectroscopy [21]. The proposed mechanism, involving multiple pathways that facilitate electron and light energy transfer, plays a positive role in optimizing biotic–abiotic hybrid systems. Therefore, establishing a robust interface and efficient intracellular transfer between artificial materials and natural cells are crucial for enhancing the utilization of external photo-induced electrons and promoting biotransformation in these biohybrids.

Bazan and co-workers have developed novel organic conjugated molecules with acceptor-donor-acceptor (A-D-A) core structures to establish an artificial N_2_ fixation system with *Azotobacter vinelandii* [93]. These molecules, when anchored to the bacterial membrane and incorporated into lipid bilayers, facilitate ET by connecting redox proteins within the membrane. This enhancement in electron flow significantly improves the efficiency of the N_2_ fixation pathway, resulting in a 2.4-fold increase in light-driven ammonia production. Improving the efficiency of light capture and photo-induced charge separation is crucial for enhancing the overall performance of the system. Yu *et al*. have developed donor-acceptor (D-A) CPNs that encapsulate poly[2-methoxy-5-(2-ethylhexyloxy)-1,4-phenylenevinylene] (MEH-PPV) and poly(fluorene-alt-thienopyrazine) (PFTP). These nanoparticles effectively harvest visible light due to their broad absorption spectra and matched energy levels, resulting in higher photocurrent [24]. The integration of these D-A CPNs into a biohybrid system led to a 37% increase in solar-driven threonine production. Moreover, through covalent attachment of threonine deaminase to the surface of the nanoparticles, efficient conversion of threonine to 2-oxobutyrate was accomplished, with stable operation for 9 days. Additionally, a compatible biotic–abiotic interface can be established through both biological modifications and the development of artificial materials. For example, engineered *Escherichia coli (E. coli)* biofilms undergo *in situ* mineralization to form photocatalytic biomembranes embedded with CdS nanoparticles [94]. Integrating enzymes or microbial cells into these biomembranes improves the overall photocatalytic efficiency.

Using gene editing to precisely modify essential bacterial metabolic pathways allows them to effectively engage in biotransformation and convert simple substrates into high-value products [95–97]. Furthermore, Joshi’s group genetically modified *Saccharomyces cerevisiae* and employed indium phosphide (InP) nanoparticles on its surface [98]. Through this modification, an energy harvesting and conversion interface was created, enabling the yeast to efficiently utilize photogenerated electrons, thereby enhancing the production of key metabolites like shikimic acid. Recently, Yuan *et al*. developed a spatiotemporal solar-decoupled biohybrid strategy [99]. This strategy is based on a persistent photocatalyst combined with photoautotrophic microbes *Rhodopseudomonas palustris* (*R. palustris*), and the resulting hybrid system achieves an apparent photoconversion efficiency of 8.30%. This value is significantly higher than the 4.36% observed in the bare *R. palustris* control group. Besides, they proposed a light-driven bionic system (*R. palustris*-ZGGO: Ni@PDA), which comprises three core modules: the photoinduced electron module, the ET channel module and the proton gradient module [100]. Within this cellular factory, the simultaneous and controllable supply of NADPH and ATP can be achieved, thereby enabling highly efficient CO_2_ fixation and biomanufacturing. By designing a dual-channel energy pathway from thylakoid to *E. coli* combining energy molecule supply and ET, Tang *et al*. developed an *E. coli*–thylakoid hybrid with a boosted ATP value and NADPH/nicotinamide adenine dinucleotide phosphate (NADP^+^) ratio for an impressive H_2_ production rate [101]. Furthermore, using the ocean carbon sink as a resource, Gao’s group proposed an electro-bioconversion system, which could serve as a viable pathway to capture and convert oceanic CO_2_ into drop-in biochemicals via decoupled electro-biocatalytic processes [102].

The advanced artificial biohybrid systems, with improved biotransformation efficiency, offer diverse compositions and enhanced functionality. Yu *et al*. designed an artificial photosynthetic symbiont by integrating multiple organisms (*Syn, R. palustris, Bacillus licheniformis* (*B. licheniformis*)) with the conducting polymer PFP (Fig. 5a and b) [30]. This system efficiently utilizes CO_2_ and N_2_ to synthesize valuable products, including *γ*-polyglutamic acid and bacitracin A, by optimizing photosynthetic processes and photoelectron production. In a similar vein, Liu’s group successfully fulfilled the conversion of CO_2_ to acetate in *S. ovata* cells by introducing external photo-induced electrons [103]. Subsequently, the generated acetate was engaged in the biosynthesis process of high-value pharmaceuticals through a material exchange cascade between *S. ovata* and genetically engineered *E. coli*. Furthermore, bacterial cells can be strategically employed to regulate ET in biohybrids for efficient biotransformation. In this regard, Reisner’s group utilized *Geobacter sulfurreducens* KN400 cells, which possess multiheme c-type cytochromes (c-Cyts) and electrically conductive protein filaments made from polymerized c-Cyts [104]. These distinctive features allowed KN400 cells to optimize the interface between a cyanamide-functionalized carbon nitride polymer (^NCN^CNx) and *Methanosarcina barkeri* to allow cooperative interaction between two microbes via direct interspecies electron transfer, thereby facilitating the biosynthesis of CH_4_ from CO_2_.

**Figure 5.**
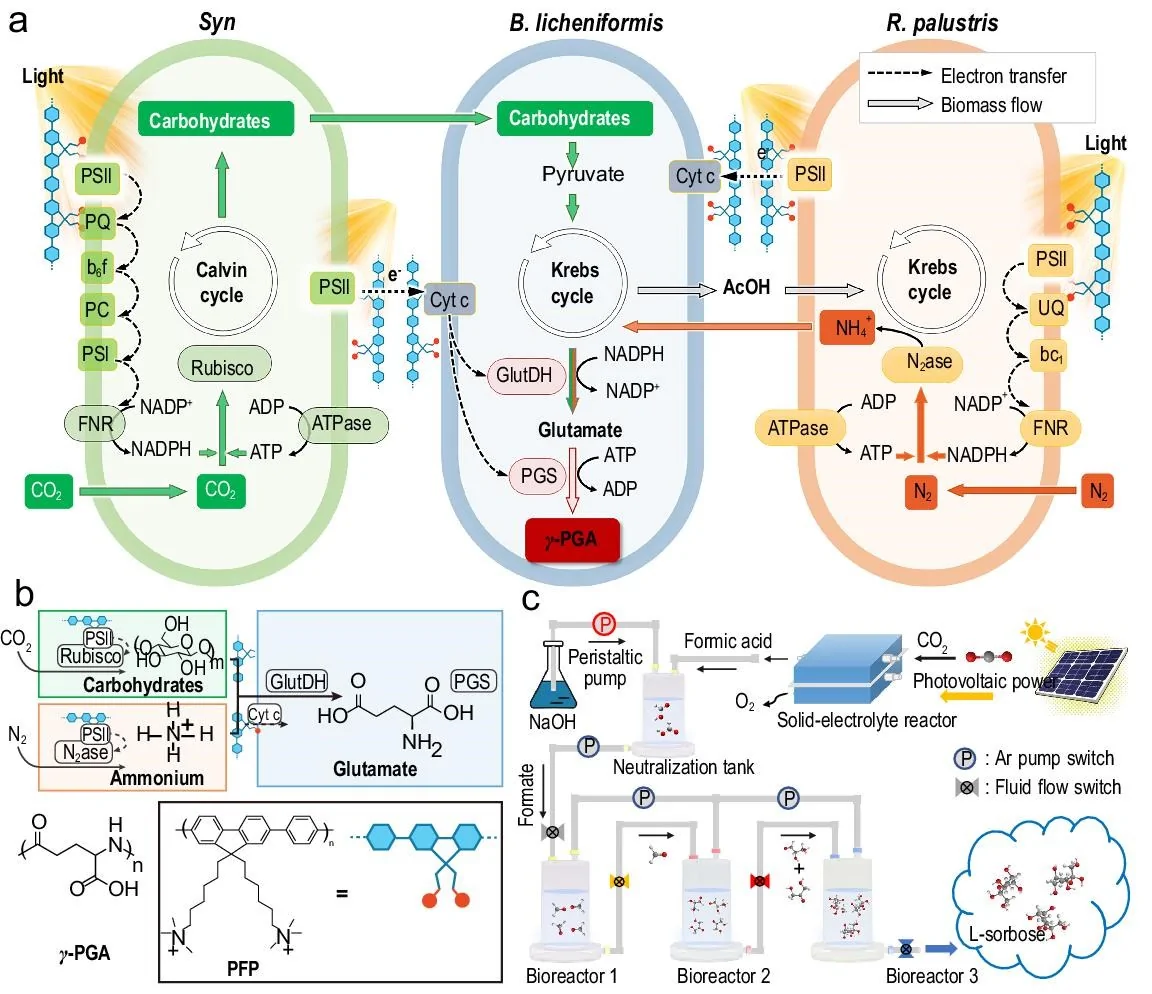
(a and b) The artificial photosynthetic symbiont by integrating multiple organisms (*Syn, R. palustris, B. licheniformis*) with the conducting polymer PFP. Adapted with permission from [30]. Licensed under CC BY 4.0. (c) The photovoltaic-powered electrocatalysis with a genetically engineered five-enzyme cascade platform. Adapted with permission from [107]. Licensed under CC BY 4.0.

Numerous studies have demonstrated that hybrid systems composed of artificial materials and biological cells can achieve efficient biotransformation. In particular, charge transfer between artificial components and biocatalysts has mainly been described by three pathways: mediation by redox-active proteins, direct electron injection into cells via membrane-associated materials and ET mediated by small-molecule shuttles. However, existing studies do not fully explain how external stimuli couple with intracellular metabolism, which remains a key challenge in this field.

### Advancing biohybrid systems through interdisciplinary integration with electrochemical devices

The development of macroscopic device architectures provides a practical route for implementing biohybrid systems in efficient biotransformation. Integrating artificial components into reusable platforms further enhances system practicality by enabling continuous operation and improved energy utilization. In these device-based strategies, energy conversion and catalytic processes are organized in two distinct ways. Photoelectrochemical (PEC) systems integrate light harvesting and catalytic reactions within a single architecture, enabling direct utilization of photogenerated charge carriers. In contrast, photovoltaic-driven electrocatalytic systems separate light absorption from catalysis by converting solar energy into electricity to drive electrochemical transformations. This fundamental difference in energy conversion pathways leads to distinct design constraints for optimizing efficiency.

For PEC systems in particular, conventional configurations coupling cathodic CO_2_ reduction with anodic oxygen evolution reactions (OER) suffer from high thermodynamic and kinetic energy demands, which limits overall efficiency and scalability. To address this bottleneck, Yang’s group has developed a photochemical diode device that utilizes red light (740 nm) to drive both CO_2_-to-multicarbon conversion and glycerol oxidation, addressing the energy limitations associated with the OER [105]. This system efficiently reduces CO_2_ to acetate while simultaneously converting glycerol into valuable oxidation products by integrating a p-type SiNW photocathode with *S. ovata* and a Pt-Au-loaded n-type SiNW photoanode. Biohybrid systems can be integrated with photovoltaic-driven electrocatalytic platforms to enable programmable control over electrochemical reactions and improve overall energy conversion efficiency. This integration also provides opportunities for coupling multiple catalytic steps, thereby enhancing product diversity and selectivity while streamlining reaction pathways. Following this strategy, Zheng *et al*. developed a hybrid system combining a CO_2_ electrolysis solid-electrolyte reactor with genetically engineered *S. cerevisiae*. This system enables the conversion of CO_2_ into high-purity acetic acid, which is subsequently utilized by engineered yeast to produce glucose and fatty acids [106]. Xiong’s group combined photovoltaic-powered electrocatalysis with a genetically engineered five-enzyme cascade platform. This system efficiently converted CO_2_ into C6 sugar (*L*-sorbose) with a solar-to-food energy conversion efficiency of 3.5%, which is >3 times higher than natural photosynthesis (Fig. 5c) [107]. Additionally, Hann *et al*. harnessed the advantages of the catalytic cascade strategy by utilizing electrochemically generated acetate as a carbon and energy source to support a variety of organisms, including algae, yeast, fungi and crops such as lettuce, rice, cowpea, tomato and *A. thaliana* [18]. By utilizing electrical energy generated within biological systems, this approach offers a more eco-friendly solution for chemical production, reducing reliance on external power sources. Chen *et al*. developed a self-powered biohybrid system using microbial fuel cells composed of conjugated polymers poly[3-(3′-*N,N,N*-triethylamino-1′-propyloxy)-4-methyl-2,5-thiophene chloride] (PMNT) and *Shewanella oneidensis*. This system utilizes bio-generated electricity to drive CO_2_ fixation for the continuous production of PHB within bacterial cells [108].

These accomplishments highlight the potential of high-efficiency biotransformation hybrid systems for sustainable chemical production. The biohybrid systems combine the light-harvesting capabilities of artificial materials with the catalytic efficiency of biological systems. The integration of artificial molecular chemistry and bioengineering plays a vital role in optimizing biotransformation efficiency and enhancing the system’s functionality. Notably, key innovations in electrode design and coupling strategies enable efficient ET, thereby improving chemical production.

## ARTIFICIAL PHOTOSYNTHESIS SYSTEMS FOR BIOTRANSFORMATION PROCESSES

### Construction and mechanisms of artificial photosynthesis systems

Following the biological blueprint of natural photosynthesis, AP, typically containing synthetic light-harvesting units and reaction centers, aims to convert H_2_O, CO_2_ and N_2_ to storable/transportable fuels or valuable chemicals [6,28,109–111], by using sunlight as the mere energy source (Fig. 6). Because of its great potential to solve both energy crises and environmental pollution simultaneously, the establishment of highly efficient and stable AP devices has been the ultimate goal of the scientific community. In a typical AP system, three main steps are included: (i) the light absorption to generate energetic excitons, (ii) the dissociation of excitons into electrons and holes and their subsequent migration to active sites and (iii) the surface redox reactions to evolve corresponding products. In fact, the concept of photo-driven chemical synthesis was proposed by G. Ciamician as early as 1912 [112], while the term ‘artificial photosynthesis’ has been generally accepted since 1972 after the seminal work of PEC H_2_O splitting using TiO_2_ by Fujishima and Honda [113]. During the past 50 years, exciting materials science, fundamental understanding of chemical reactions, advanced characterization techniques and device design and engineering have been significantly developed with great enthusiasm, which has established a solid foundation for the practical applications of AP systems.

**Figure 6.**
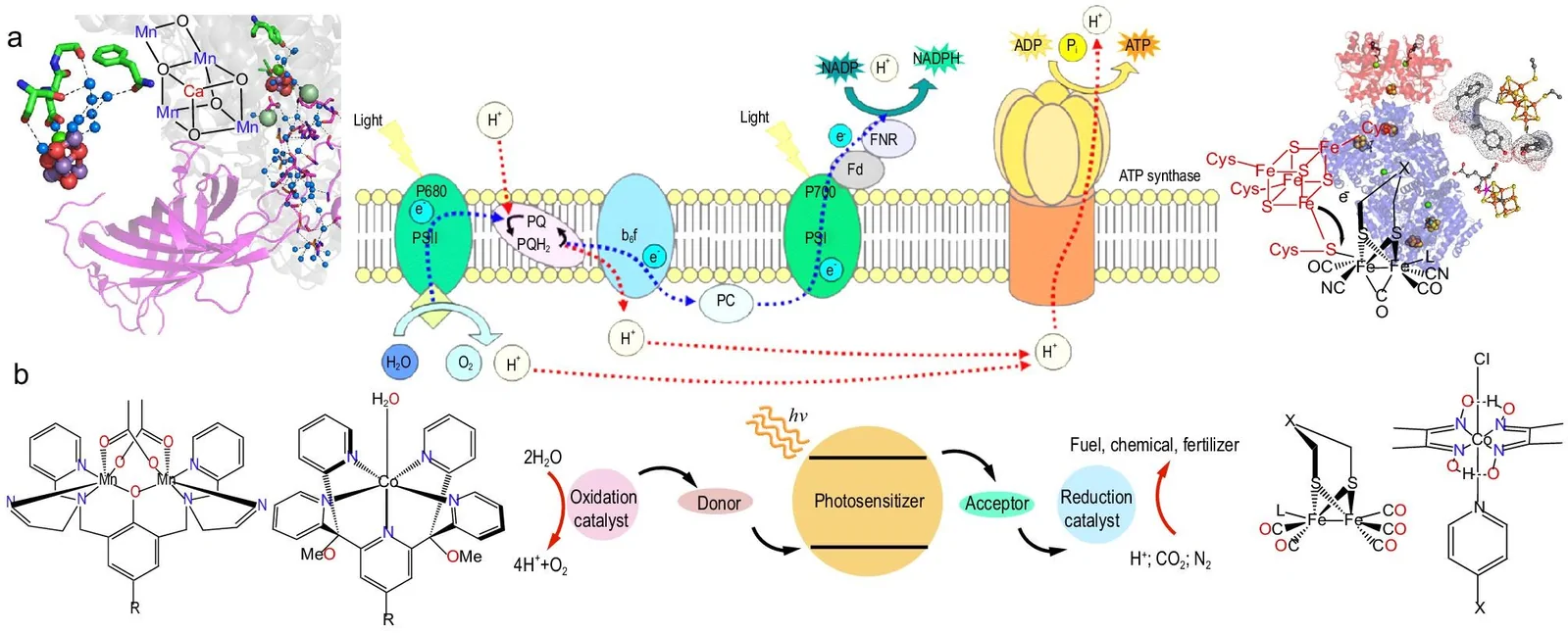
The use of AP for solar to chemical conversion. (a) Schematic representation of the natural photosynthetic chain in photosystems and corresponding enzymes. (b) The basic working mechanism of artificial photosynthesis. Adapted with permission from [28,109–111]. Copyright 2022, American Chemical Society. Copyright 2017, American Chemical Society. Copyright 2019, American Chemical Society. Copyright 2015, American Chemical Society.

### Artificial photosynthesis systems for beyond natural synthesis

Recent advances in AP research have increasingly underscored the significance of its great potentials in H_2_ production, CO_2_ reduction, N_2_ fixation and beyond. In nature, the OEC oxidizes H_2_O with a low overpotential and a high rate of 100–400 s^−1^ under ambient conditions, while [FeFe]-hydrogenase with binuclear Fe–Fe cluster evolves H_2_ with remarkable catalytic activity (6000–9000 s^−1^). Inspired by biological models, a series of metal complex mimics based on Ru, Mn, Cu, Fe, Co and Ni have been established for H_2_O oxidation or H_2_ evolution by Meyer, Thummel, Sun, Wu, Zhang and others [52,53,114–116]. The great achievement in bioinspired metal complexes with clear structure–activity relationship sheds light on the mechanistic understanding and new photocatalysts’ design. Meanwhile, various semiconductors, including metal oxide, (oxy)nitrides, nitrides and sulfides consisting of metals with either *d*^0^ or *d*^10^ configurations, have been verified as effective photocatalytic materials for overall H_2_O splitting by Domen, Nocera, Li, Zou and others [117–120]. For instance, it is revealed that the modified aluminum-doped strontium titanate (SrTiO_3_:Al) photocatalyst can split H_2_O to H_2_ and O_2_ with an internal quantum efficiency of almost unity under ultraviolet (UV) light. Recently, organic semiconductors [121], especially C_3_N_4_ and covalent organic frameworks, have been confirmed as effective counterparts for photocatalytic H_2_O splitting. For instance, Shen *et al*. realized stoichiometric H_2_ and O_2_ evolution with a solar-to-hydrogen efficiency up to 1.16% by using 2D/2D C_3_N_4_ polymeric heterostructure [122].

For efficient, stable and highly selective CO_2_ reduction, scientists have conducted extensive research on the construction of homogeneous or heterogeneous artificial photosystems. For instance, a series of metal complexes by Wu *et al*. [123] and Robert *et al*. [124], and dinuclear metal synergistic complexes by Lu *et al*. [125] are synthesized for artificial photosynthetic CO_2_ reduction. By mimicking the natural photosynthetic apparatus, Tian *et al*. constructed an artificial spherical chromatophore nanomicelle from Zn porphyrin amphiphiles with a Co catalyst, which realized efficient CO_2_-to-CH_4_ conversion in water [126]. These works underline the importance of the coordination environment, metal–metal synergy and spatial sphere structure in regulating CO_2_ activation and reduction pathways. In the scenario of heterogeneous photosystems, Deng *et al*. created ‘molecular compartments’ inside metal-organic framework (MOF) crystals by growing TiO_2_ nanocrystals, which realizes CO evolution with an apparent quantum efficiency of 11.3% at 350 nm [127]. Wu *et al*. coupled CO_2_ reduction with organic synthesis under solar light using quantum dots (QDs) photocatalysts, providing an exact answer to low-cost and effective CO_2_ valorization [128,129]. Expanding the utilization of AP in N_2_ fixation is extensively studied, through which ammonia can be obtained via the multielectron and multiproton photochemical processes [28]. The synergistic activation of N_2_ with other small molecules is also reported. For example, Zhang *et al*. disclosed an effective strategy of accelerating ET dynamics by TiO_2_-immobilized reversible single-atom copper to enhance the performance of urea photosynthesis from N_2_, CO_2_ and H_2_O [130]. Artificial photosynthetic N_2_ fixation is still in its early stages, and there is huge space to improve the reactivity and durability.

### Multifield sequential cascade and scale-up of the artificial systems

Through years of development, the potential application of AP for scale-up mass transformation already shows the signs. In 2021, Domen *et al*. reported the safe operation of a 100 m^2^ array of panel reactors over several months with autonomous recovery of H_2_ from the moist gas product mixture by a commercial membrane (Fig. 7a) [131]. In 2022, Reisner *et al.* indicated that the floating perovskite-BiVO_4_ devices showed great potential for scalable solar fuel production by using sunlight (Fig. 7b) [132]. By integrating a multifield sequential cascade, Li *et al*. established a ‘liquid sunshine’ project for the commercial production of green alcohols (Fig. 7c) [133]. In the scale-up utilization of artificial photosynthetic systems, achieving the massive preparation of photocatalysts under mild conditions and addressing the issues of photocatalysts deactivation and recovery are prerequisites for their practical applications. More importantly, compared to laboratory-scale systems, scale-up applications of artificial photosystems involve orders of magnitude increase in macroscopic dimensions, thus facing the challenge of limited mass and energy transfer. How to couple internal fields (such as built-in electric fields) with external fields (such as light fields) at the macroscopic scale to overcome the challenges of low efficiency and poor directionality in mass and energy transfer will be the key points for realizing the practical application of AP. As discussed, both opportunities and challenges exist in this field of AP. What we can do is always believe that something wonderful is about to happen. In the future, multidisciplinary collaborations among different subjects will be of utmost significance to contribute to the rational design and construction of AP for H_2_ evolution, CO_2_ reduction, N_2_ fixation and beyond.

**Figure 7.**
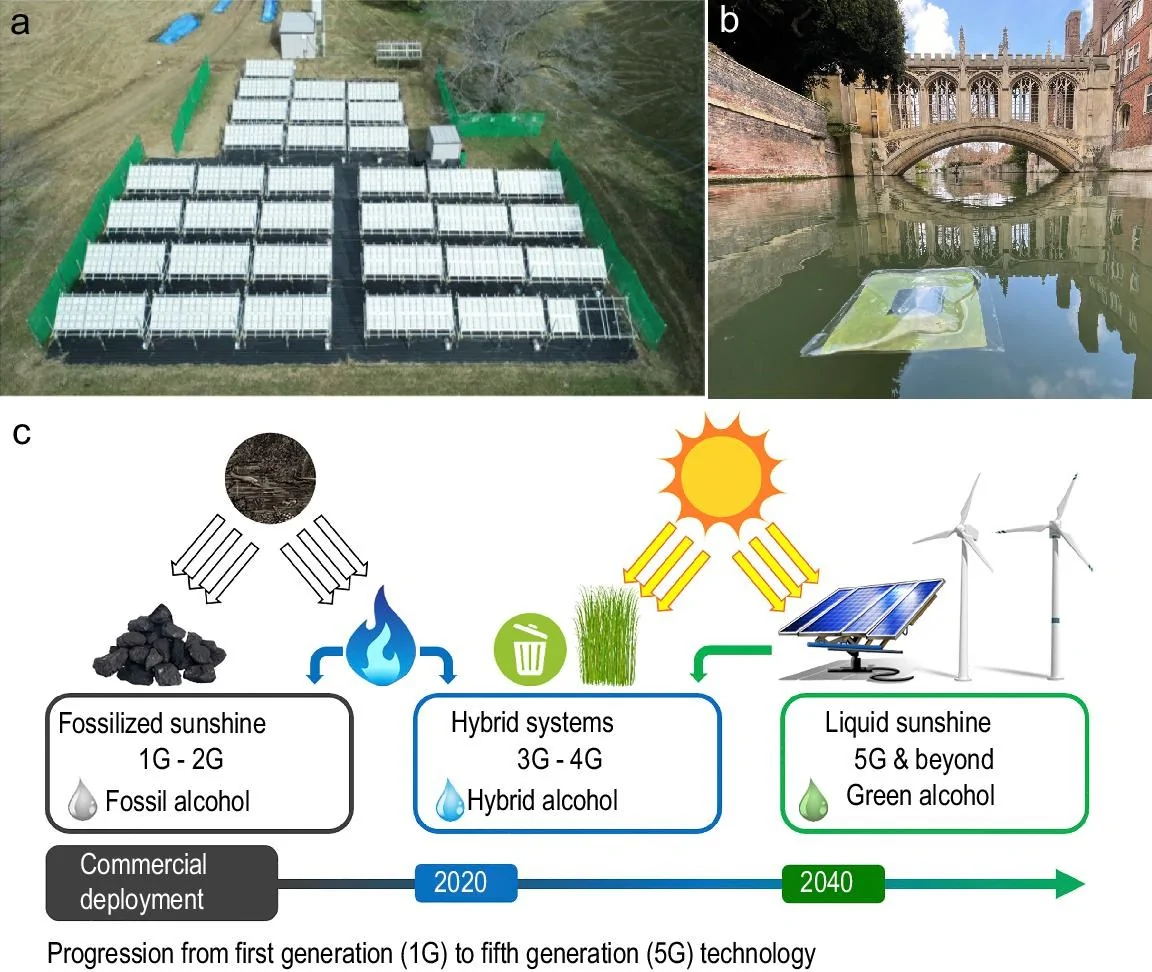
Representative examples showing the practical applications of AP. (a) Overall water splitting panels. Adapted with permission from [131]. Copyright 2021, Springer Nature. (b) PEC artificial leaves for outdoor solar fuels production. Adapted with permission from [132]. Copyright 2022, Springer Nature. (c) Liquid sunshine roadmap. Adapted with permission from [133]. Copyright 2018, Elsevier.

## SUMMARIES AND PROSPECTS

Biotransformation plays a pivotal role in addressing global resource and energy challenges, yet inefficiencies, slow reaction kinetics and limited product diversity often constrain natural processes like photosynthesis and microbial metabolism. Many breakthroughs have expanded the potential of biotransformation, enabling scalable solutions for CO_2_ reduction, nitrogen fixation and the sustainable production of high-value chemicals. By integrating insights into the static structures and dynamic mechanisms of complex biological systems, along with photosynthesis-inspired approaches, significant progress has been made in diversifying functional outputs (Table 1). Moving forward, addressing persistent challenges and exploring novel strategies will be essential to unlocking the full potential of biotransformation in sustainable chemical and energy production.

**Table 1.** Summary of solar-driven artificial hybrid systems.

Hybrid systems	Products	Production yield	Quantum yield	Enhancement factor	Refs
*C. pyrenoidosa*/PBF	Dry cell weight (DCW)	1.118 g L^−1^	N.A.	1.15	[12]
PDI/PFP/*M. thermoacetica*	CH_3_COOH	0.63 mM	1.60%	6.3	[60]
CdS-NPs/*M. thermoacetica*	CH_3_COOH	1.3 mM	2.44%	N.A.	[20]
CdS:MoFe protein	NH_3_	500 nmol NH_3_ (mg MoFe protein)^–1^ min^–1^	3.3%	N.A.	[19]
PFP/*A. chroococcum*	NH_3_	1.40 μg/10^9^ cell	0.83%	1.44	[91]
InP QDs/*S. ovata*	CH_3_COOH	0.89 mmol L^−1^ h^−1^	6%–8%	N.A.	[23]
PFODTBT Pdots/*R. palustris*	PHB	21.3 ± 3.78 mg L^−1^	2.27%	2.92	[92]
COE-IC/*A. vinelandii*	NH_3_	20.6 ± 0.4 nmol mL^−1^	N.A.	2.6	[93]
*E. coli*/D-A CPNs@Enzyme	2-oxobutyrate	6.0 ± 0.15 mM (96 h)	1.25%	1.6	[24]
*S. cerevisiae*-*InP*	Shikimic acid	48.5 ± 2.1 mg L^−1^	1.58%	24	[98]
ZGGO:Ni/*R. palustris*	Lycopene	8.80 mg L^−1^	8.30%	1.7	[99]
*E. coli*–thylakoid	H_2_	15.1 mmol h^−1^ g^−1^_dcw_	N.A.	2	[101]
Electro-bioconversion system	Succinic acid	1.37 g L^−1^	N.A.	N.A.	[102]
*Syn*/*R. palustris*/*B. licheniformis*/PFP	*γ*-PGA	144.2 mg L^−1^	1.43%	13.5	[30]
*KN400/G. sulfurreducens + M. barkeri*	CH_4_	110 μmol g_CNx_^−1^	N.A.	5.5	[104]
Electrocatalytic−biocatalytic flow system	*L*-sorbose	105.0 mg L^−1^ h^−1^	3.5%	N.A.	[107]
MOF-based ‘molecular compartments’ artificial photocatalysts	CO	12 mmol g^−1^ h^−1^	11.3%	1.4 × 10^4^	[127]
ZnSe/CdS dot-on-rod photocatalytic device	CO	11.3 μmol g^−1^ h^−1^	N.A.	N.A.	[129]
100 m^2^ water splitting photocatalyst panel reactor	H_2_	N.A.	0.76% (STH)	N.A.	[131]

With the advancement of various biophysical and biochemical techniques, significant progress has been made in understanding the mechanisms underlying natural solar-driven reactions and biotransformation processes. However, there are also several open questions to be answered. First, the exact reaction mechanism underlying crucial steps in photosynthesis, as well as possible structural rearrangements during the S-state transitions, remains unclear due to the absence of the structures of PSII in more intermediate S states. Second, monitoring the ET pathways within the megacomplex of photosynthesis is still challenging. Third, natural biotransformation processes often involve the collaborative actions of a series of enzymes. Current studies focusing solely on a single enzyme or a limited number of enzymes are inadequate to reveal the core mechanisms of biotransformation from a holistic perspective. It is necessary to investigate the catalytic mechanisms of enzymes in a more native environment. Last, new techniques are needed to characterize the new mechanism for newly developed hybrid systems, which would benefit the development of better solar-driven artificial hybrid systems.

As a burgeoning field, some current research primarily focused on discovering new-to-nature reactions and exploring non-natural chemical transformations. Advancements in protein *de novo* design and artificial intelligence (AI) are opening up exciting prospects for creating enzymes with customized reactivities. However, several key challenges remain, particularly in the rational design of enzymes and artificial catalysts that are compatible with photochemical or electrochemical environments. A clearer mechanistic understanding of these coupled catalytic systems is also needed. Such insights are essential for system optimization and predictive control. In addition, despite the promise of these proof-of-concept studies, a significant gap remains between laboratory-scale discovery and practical application. This gap arises from limited catalytic efficiency and productivity, as well as challenges in scale-up. Overcoming these scale-up challenges requires advances in bioprocess and reactor engineering, together with data-driven approaches such as AI for process optimization and predictive control. These challenges highlight the need for interdisciplinary collaboration across chemistry, biology and engineering.

Furthermore, while both enzymatic and microbial hybrid systems share common challenges in efficiency, stability and interfacial ET, they diverge significantly in their operational constraints. Enzyme-based systems require precise control over ET distances and cofactor compatibility, with particular sensitivity to photodamage under UV/violet light. In contrast, bacteria-based systems must overcome metabolic strain and oxygen sensitivity (e.g. nitrogenase), alongside establishing efficient transmembrane electron transport pathways. Future strategies must therefore adopt distinct optimization approaches: *de novo* protein design and AI for enzyme engineering versus metabolic engineering and biointerface optimization for bacterial systems.

To enhance the photosynthetic efficiency of biohybrid systems, we should prioritize strategies that optimize light absorption, photoelectric conversion and interfacial ET. The use of broad-spectrum materials can effectively expand the absorption range, while structured assemblies, such as nanoarrays, offer a promising approach to improving light harvesting. It is also important to consider biological constraints, including nanomaterial toxicity, metabolic burden and genetic instability. Beyond these considerations, achieving high efficiency further requires precise control over the physicochemical processes that govern ET. In particular, aligning the energy levels of semiconducting materials with biological ET chains is essential to maximize efficiency. Strengthening interfacial interactions through targeted chemical or physical modifications can further reduce resistance, thereby facilitating ET and enhancing photoelectric conversion. In addition, integrating artificial molecular chemistry with bioengineering represents a key strategy for improving biotransformation efficiency. Future efforts should focus on the design of advanced electrodes and the optimization of coupling mechanisms to enable efficient ET and enhance chemical production.

For practical applications, scalable and sustainable biohybrid systems require not only performance optimization but also evaluation of material recyclability and environmental safety. Artificial components must overcome key challenges, including cytotoxicity, metabolic interference and instability in biological environments. From a materials design perspective, organic conjugated molecules offer a promising solution, as their carbon-based frameworks are intrinsically compatible with biomacromolecules and support efficient interfacial interactions. Their molecular tunability enables precise control of energy levels and hydrophobicity, improving charge transfer and spatial localization. In addition, structural design allows potential biodegradability, enabling closed-loop material use. Future research should prioritize the development of advanced recycling technologies and modular, device-based systems. These advances will help address current limitations and guide the development of next-generation biohybrid systems.

Recent interdisciplinary advancements have driven the development of innovative solar-driven artificial hybrid systems, which have begun to transcend the inherent limitations of natural bioconversion processes. These systems not only achieve efficiencies and functionalities that partially surpass those of natural systems but also exhibit promising advantages in scalability, cost-effectiveness and environmental sustainability. Importantly, the integration of AI and machine learning is emerging as a powerful enabler in this field, offering data-driven strategies to predict photoenzyme reactivity, optimize semiconductor–bio interfaces and guide the rational design of hybrid architectures. By accelerating the identification of key parameters governing ET and metabolic flux distribution, AI-assisted approaches are expected to further enhance system performance and facilitate the transition from empirical optimization to predictive and programmable design.

Such progress underscores their potential to revolutionize industrial applications, particularly in areas such as carbon dioxide reduction, nitrogen fixation and the green synthesis of value-added chemicals. We are confident that further integration of insights from biology, chemistry, materials science and engineering will foster groundbreaking advancements in this field. The continued exploration of solar-driven artificial hybrid systems is expected to extend its impact across diverse domains, promising an even broader and brighter future.
